# Microbe Profile: *Streptomyces coelicolor:* a burlesque of pigments and phenotypes

**DOI:** 10.1099/mic.0.000821

**Published:** 2019-08-01

**Authors:** Justin R. Nodwell

**Affiliations:** ^1^ Department of Biochemistry, University of Toronto, 661 University Avenue, Toronto, ON M5G 1M1, Canada

**Keywords:** *Streptomyces coelicolor*, sporulation, specialized metabolism, cell division

## Abstract

The streptomycetes are soil-dwelling bacteria that are found in soil everywhere on Earth: the molecule geosmin, which they produce as part of their life cycle, is what gives soil its familiar ‘earthy’ smell. The species is best known for the production of biologically active small molecules called ‘natural products’. These molecules are the source of most of our antibiotics and anti-fungals, as well as many other drugs. The streptomycetes have a filamentous form rather than the more familiar rod-shaped spirochete and coccoid forms. They exhibit a complex life cycle and sporulation mechanism involving several differentiated cell types, each having specific roles in the colony life history. *Streptomyces coelicolor* is an important model system for this genus – research on this bacterium has provided foundational information for all of these fascinating processes.

## Taxonomy

Phylum, *Actinomybacteria*; class, *Actinobacteria *; order, *Actinomycetales *; family, *Streptomycetaceae *; genus, *Streptomyces *; species, *Streptomyces coelicolor* A3(2) – see http://StrepDB.streptomyces.org.uk. Note that this species is distinct from *S. coelicolor Müller ATCC 23899*.

## Properties

*S. coelicolor* spores germinate and propagate ‘substrate hyphae’ that grow by tip extension and branching. Substrate hyphae generate cross-walls at sporadic locations but do not divide; many chromosomes share each cross-wall-bounded compartment. A second filamentous cell type, the ‘aerial hyphae’, grows into the air, conferring a white, fuzzy appearance to the colony surface. In contrast to the substrate hyphae, aerial hyphae undergo a concerted round of septation that divides them into compartments, each having one chromosome. These compartments then develop into spores. Many steps in this life cycle are marked by brilliant pigmentation. Thus, the substrate hyphae produce blue (actinorhodin), red (undecylprodigeosin) and yellow (coelimycin) specialized metabolites, while the completion of spore maturation is marked by the deposition of a grey pigment in the spore walls. Genetic analysis of these pigmentation phenomena facilitated the discovery of many of the genes that control the *S. coelicolor* life cycle.

## Genome

The genome of *S. coelicolor* (strain M145) is a linear, 8 667 507 bp sequence with 7825 genes. The core 4.4 Mbp shares synteny with other actinobacteria, such as *Mycobacteria* and *Corynebacteria*. Terminal arms of 1.5 Mbp (left) and 2.3 Mbp (right) are more variable and less dense in terms of gene content and encode many biosynthetic genes for specialized metabolites. The linear *S. coelicolor* chromosomes have inverted repeats that function as telomeres that are bound by a telomere-binding protein that facilitates end replication [1]. In addition, *S. coelicolor* has two episomes: SCP1, a linear molecule of 356 023 bp, and SCP2, a circular molecule of 31 317 bp. Both are mobile genetic elements.

## Phylogeny

Common laboratory strains of *S. coelicolor* include M600 and M145 (which lacks SCP1 and SCP2). Strains M1152 and M1154 (both derived from M145) have been developed as heterologous hosts for secondary metabolites.

## Key Features and Discoveries

### Growth, cell division and morphogenesis

Filamentous growth uses the same core machinery as unicellular bacteria for cell wall biosynthesis and cell division, although it is deployed and regulated very differently. The DivIVA protein is localized to the tips of growing cells, where it is believed to organize proteins involved in cell wall growth and branching [2]. DivIVA is subject to serine phosphorylation by the AfsK kinase when the wall is damaged by compounds such as bacitracin – this modification drives the branching of vegetative hyphae [3]. Cytoskeletal proteins such as FilP polymerize to form intermediate filaments involved in hyphal integrity [4].

Cell division genes such as *ftsZ* are expressed at a low level in substrate hyphae. Flärdh and co-workers discovered a developmentally regulated promoter that drives elevated expression of these genes exclusively in the aerial hyphae so as to activate the sporulation septation events that lead to spore formation. *Streptomyces *-specific SALP proteins such as SsgB are thought to coordinate the formation of the sporulation divisome [5]. McCormick and co-workers showed that most cell division genes are dispensable for viability. Slow-growing, non-sporulating null mutants have since been generated in *ftsZ* and, indeed, all of the cell division genes.

Specialized proteins allow aerial hyphae to break the air–water interface and grow away from the colony surface. One of these is SapB, a 22 amino acid surfactant that is chemically related to the lantibiotic antimicrobials [6]. Another class, the functional amyloids known as chaplins, forms an intricately structured sheath that covers each aerial filament [7, 8].

### Secondary metabolism

Research on the production of the antibiotic actinorhodin led to the demonstration by Malpartida that the actinorhodin biosynthetic pathway is encoded in a discrete cluster of all the genes that are necessary for production, export and self-resistance. The cluster also encodes the *actII orf4* gene, which encodes a cluster-specific transcription factor that activates the genes in the cluster. Higher order or pleiotropic regulatory mechanisms directly control the expression of this activator. There are at least 27 biosynthetic gene clusters for specialized metabolites in the *S. coelicolor* genome; cluster-specific and pleiotropic regulation are shared features of many of them. Indeed, it is likely that these themes are universal in the genus *Streptomyces *.

### Regulatory mechanisms

*S. coelicolor* exhibits a remarkable array of regulatory mechanisms that govern its life cycle. Lawlor and co-workers demonstrated that *bldA* encodes a leucyl tRNA that is required for the translation of a rare UUA codon found in several developmental regulators. BldD, a transcriptional master regulator that controls the cell type-specific expression of some sporulation genes, is held in dimeric form via an interface composed entirely of four molecules of cyclic-di-GMP [9], a unique use of this signalling molecule. γ-butyrolactone signalling molecules and at least 15 pleiotropic regulators have been shown to regulate the expression of the various pigmented specialized metabolites. Genetic and chemical manipulation of these regulatory pathways has been exploited to help mine for new antibiotics [10].

Another important regulatory mechanism that was discovered by Lonetto and is important in many bacterial species is ‘extracytoplasmic function’ (ECF) σ factors. These are repressed by a membrane-associated anti-sigma factor until an appropriate signal triggers release so that the σ can direct the expression of signal-responsive genes.

## Open Questions

There are many important questions in *Streptomyces* biology – *S. coelicolor* will continue to play an role in their solution, even as faster-growing models such as *Streptomyces venezuelae* come into use.

- Why does *S. coelicolor* have so many transcription factors?
- What distinguishes vegetative cross-walls from sporulation septa?
- How are linear chromosomes organized, replicated and segregated?
- What is the full set of roles played by the *S. coelicolor* specialized metabolites?
- Do the cell–cell signals produced by streptomycetes act as quorum sensors?
